# Interleukin-37 Inhibits Interleukin-1β-Induced Articular Chondrocyte Apoptosis by Suppressing Reactive Oxygen Species

**DOI:** 10.3390/biomedicines12092025

**Published:** 2024-09-04

**Authors:** Seong-Kyu Kim, Boyoung Kim, Jung-Yoon Choe, Ji-Won Kim, Ki-Yeun Park

**Affiliations:** 1 Division of Rheumatology, Department of Internal Medicine, Catholic University of Daegu School of Medicine, 33, Duryugongwon-ro 17-gil, Nam-gu, Daegu 42472, Republic of Korea; 2 Arthritis and Autoimmunity Research Center, Catholic University of Daegu, 33, Duryugongwon-ro 17-gil, Nam-gu, Daegu 42472, Republic of Korea

**Keywords:** osteoarthritis, chondrocyte, interleukin-1β, interleukin-37, apoptosis, reactive oxygen species

## Abstract

**Objective**: Chondrocyte apoptosis has been considered a crucial mechanism that is responsible for cartilage destruction in osteoarthritis (OA). The mechanism of interleukin-37 (IL-37) on chondrocyte apoptosis has not been clearly determined in the pathogenesis of OA. Here, we explored the role of IL-37 in the regulation of cellular apoptosis in rat chondrocytes stimulated by IL-1β. **Methods**: Rat chondrocytes were used in in vitro study, and were stimulated with IL-1β (10 ng/mL) and/or recombinant IL-37 (rIL-37; 100 ng/mL) after cytotoxicity assessments using these cytokines were conducted. After rIL-37 treatment of chondrocytes stimulated with IL-1β, the cell proliferation assay, apoptosis assays, including expression of mitochondrial apoptosis-related markers, flow cytometry analysis of annexin V-FITC/propidium iodide (PI), cell cycle analysis, and Hoechst 33342 staining, and reactive oxygen species (ROS) measurement were used. **Results**: IL-1β induced expression of inflammatory cytokines and triggered degradation of the extracellular matrix of rat chondrocytes, but this effect was significantly attenuated by rIL-37 treatment. Enhanced ROS generation following IL-1β stimulation was reduced in a dose-dependent manner after stimulation with rIL-37. IL-1β induced pro-apoptotic markers and suppressed anti-apoptotic markers in rat chondrocytes. Flow cytometry using annexin V-FITC/PI revealed that IL-1β increased the apoptosis rate of rat chondrocytes, and that this effect was markedly reversed by treatment with rIL-37. **Conclusions**: IL-37 potently attenuated IL-1β-mediated apoptosis of rat chondrocytes by blocking ROS production. This study suggests that IL-37 can serve as a novel anti-cytokine therapy in OA by blocking chondrocyte apoptosis.

## 1. Introduction

Osteoarthritis (OA) is a chronic degenerative arthritis characterized by structural and functional changes in the whole joint, involving the hyaline articular cartilage, subchondral bone, meniscus, ligaments, capsule, infrapatellar fat pad, and the synovium; finally, OA leads to pain, disability, and poor quality of life [1,2,3]. Age, female gender, joint injury, and comorbidities are important risk factors for OA [3]. The pathogenic mechanism of OA is induced by biomechanical triggers, activation of inflammatory cascades, metabolic disturbance, and some genetic components, leading to loss of articular cartilage, meniscal damage, synovial inflammation, infrapatellar fat pad inflammation and fibrosis, and bony alternations [1,2,3]. Apoptosis is one of several tightly regulated mechanisms of cell death in pathological conditions such as malignancy and degenerative diseases [4]. Decreased numbers of chondrocytes, the only type of cell in mature cartilage, have been noted in OA joints, suggesting that chondrocyte apoptosis is responsible for the development of OA [5]. Dysregulated apoptosis in chondrocytes is recognized as playing an important role in the pathogenesis of OA [5,6,7].

Imbalances in biomolecular mediators, including pro- and anti-inflammatory cytokines, chemokines, and anabolic and catabolic factors, have been identified in the articular joint of OA [8,9]. Proinflammatory cytokines highly presented in interleukin-1β (IL-1β), IL-6, tumor necrosis factor-α (TNF-α), IL-8, and IL-17, contribute to the loss of articular cartilage, structural bone remodeling, and stimulation of destructive matrix-degrading enzymes [10,11,12,13]. Data from human and experimental studies provided evidence of a beneficial effect of anti-cytokine treatment using therapeutic agents targeting IL-1 and TNF in OA [14,15].

As a member of the IL-1 family, IL-37 is a novel cytokine with anti-inflammatory properties and an immunomodulatory effect that reduces inflammatory and immune responses by blocking production of proinflammatory cytokines [16,17]. IL-37 is involved in the pathogenesis of diverse autoimmune diseases and systemic inflammatory diseases, and it may be a promising therapeutic strategy against disease progression of OA [18,19,20,21]. These studies have demonstrated that IL-1β significantly induced IL-37 expression, but IL-37 suppressed the production and release of proinflammatory cytokines and proteolytic enzymes, including matrix metalloproteinases (MMPs), and the loss of proteoglycans in human chondrocytes. IL-37 mRNA and protein levels in synovial tissue and peripheral blood mononuclear cells of erosive inflammatory OA (EIOA) were much higher than those of healthy controls [22]. In addition, treatment with IL-37 in synovial cells in EIOA reduced proinflammatory cytokines. However, there is little data about the role of IL-37 in the apoptosis of chondrocytes in the pathogenesis of OA. We therefore evaluated the role of IL-37 in regulating the degradation of the extracellular matrix (ECM), release of inflammatory cytokines, and chondrocyte apoptosis in OA.

## 2. Materials and Methods

### 2.1. Cell Preparation and Culture

The rat articular chondrocyte cell line (Ref. P10930, Innoprot, Derio, Spain) was plated at a density of 5 × 10^5^/cm^2^ in T-75 flasks (Nunc, Roskilde, Denmark), and cultured in alpha minimum essential medium containing 1% L-glutamine, 1% non-essential amino acids, 1% 4-(2-hydroxyethyl)-1-piperazineethanesulfonic acid, 1% penicillin, and 1% streptomycin, supplemented with 10% fetal bovine serum.

### 2.2. Cell Proliferation Assay

Cell proliferation was measured using a Cell Counting Kit-8 (CCK-8), according to the manufacturer’s instructions (Abcam, Cambridge, MA, USA). Recombinant rat IL-1β protein (R&D Systems, Minneapolis, MN, USA) and IL-37 protein (residues 46–218) were purchased from Bio-Techne Corporation (Minneapolis, MN, USA). Cells were seeded at a density of 2 × 10^3^ cells/well into 96-well plates, and were pretreated with IL-1β at concentrations 0, 1, 5, 10, and 20 ng/mL, or recombinant IL-37 (rIL-37) at 0, 1, 10, 100, 300, and 500 ng/mL for 24 h and 48 h. The medium was removed, 10 μL of CCK-8 solution was added to each well, and cells were incubated for 1 h at 37 °C. The optical density was measured at 570 nm by a microplate reader (BMG Lab Technologies, Offenburg, Germany). Experiments were performed in triplicate.

### 2.3. Quantitative Real-Time PCR

Cells were pretreated with the indicated concentrations of rIL-37 for 24 h, and then stimulated with or without IL-1β (10 ng/mL) for 48 h. Total RNA was isolated from cell cultures using Trizol reagent (Gibco BRL, Grand Island, NY, USA) according to the manufacturer’s instructions. RNA was reverse-transcribed into cDNA using ReverTra Ace-α-1 (Toyobo, Osaka, Japan), followed by incubation at 37 °C for 15 min, 50 °C for 5 min, and 98 °C for 5 min. Quantitative real-time polymerase chain reaction (qRT-PCR) amplification was performed using a MiniOpticon Real-time PCR system (Bio-Rad, Hercules, CA, USA), and an SYBR Green Master Mix (ToYoBo, Tokyo, Japan). For PCR amplification, 2 μL of cDNA was mixed with 10 μL of SYBR Green Realtime PCR Master Mix, 10 pmol/L of primer, and 6.4 μL of RNase-free water (for a total volume of 20 μL). All reactions were performed in triplicate, and target gene expression was analyzed using the 2^−ΔΔCT^ method.

### 2.4. Western Blot Assay

After treatment, the harvested cells were washed with a phosphate-buffered saline (PBS) solution, and resuspended in a lysis buffer (Bio-Rad, Hercules, CA, USA), supplemented with a protease inhibitor (Roche Diagnostics, Mannheim, Germany), for 15 min on ice. The supernatant was collected, and protein concentrations were measured using a Pierce BCA Protein Assay Kit (Thermo Fisher, Waltham, MA, USA).

Equal amounts of total proteins (50 μg) were separated on 10–12% sodium dodecyl sulfate-polyacrylamide gel electrophoresis and transferred to nitrocellulose membranes (Bio-Rad, Hercules, CA, USA) by electrophoresis. The membranes were blocked in 5% BSA (BD Bioscience, San Francisco, CA, USA) and probed with appropriate dilutions of primary antibodies, followed by horseradish peroxidase-conjugated secondary antibodies. Finally, the proteins were visualized and enhanced using an ECL Chemiluminescence Kit (Thermo Fisher, Waltham, MA, USA).

The primary antibodies used in the experiments were as follows: anti-Bax, anti-cleaved PARP, anti-Bcl-2, and anti-β-actin from Santa Cruz Biotechnology (Santa Cruz, CA, USA); anti-IL-6, anti-collagen II, anti-aggrecan, anti-Indian hedgehog (anti-Ihh), anti-caspase3, and anti-caspase-9 from Abcam (Cambridge, MA, USA); anti-MMP-13 from MyBiosource (San Diego, CA, USA); anti-cleaved caspase-3 and anti-cleaved caspase-9 from Cell Signaling Technology (Danvers, MA, USA); and anti-TNF-α from R&D Systems (Minneapolis, MN, USA).

### 2.5. Reactive Oxygen Species Measurement

ROS were determined using a DCFDA/H2DCFDA-Cellular ROS Assay Kit (Abcam, Cambridge, MA, USA) according to the manufacturer’s instructions. Cells (1 × 10^5^) were seeded on 35 mm culture dishes and pretreated with the indicated concentrations of rIL-37 for 24 h, then stimulated with or without IL-1β (10 ng/mL) for 48 h. Finally, the cells were harvested and analyzed using a FACScan flow cytometry system (Becton Dickinson, San Jose, CA, USA).

### 2.6. Apoptosis Analysis

Apoptosis of chondrocytes was evaluated using the annexin V-fluorescein isothiocyanate (FITC)/propidium iodide (PI) double-labeling method and flow cytometry. Cells were seeded at a density of 5 × 10^5^ cells in 60 mm dishes and were pretreated with the indicated concentrations of rIL-37 for 24 h, then stimulated with or without IL-1β (10 ng/mL) for 48 h. Apoptotic cells were examined using an FITC Annexin V Apoptosis Detection Kit (BD Biosciences, San Diego, CA, USA) according to manufacturer’s protocol. Cells were harvested and resuspended in 100 μL of a 1× binding buffer. Cells were stained with an FITC-conjugated annexin V and PI labeling solution, then incubated for 15 min in the dark. After the addition of 400 μL of a 1× binding buffer, the cells were analyzed by flow cytometry using a FACScan flow cytometry system (Becton Dickinson, San Jose, CA, USA).

### 2.7. Hoechst 33342 Staining

The experimental cells were seeded at a density of 5 × 10^4^ cells in a 4-well imaging chamber, pretreated with the indicated concentrations of rIL-37 for 24 h, and stimulated with or without IL-1β (10 ng/mL) for 48 h. Cells were fixed with 4% paraformaldehyde and permeabilized using a permeabilization buffer (Invitrogen, Carlsbad, CA, USA) for 10 min at room temperature. The fixed cells were washed with PBS and stained with Hoechst 33342 (Molecular Probe, Eugene, OR, USA) for 15 min at room temperature in the dark. Stained nucleic DNA was observed under an Olympus fluorescence microscope (IX-71, Apo 100XOHR (100X, N.A.1.65), Shinjuku-ku, Tokyo, Japan).

### 2.8. Cell Cycle Analysis

Rat chondrocytes (3 × 10^5^) were seeded on 60 mm culture dishes and incubated overnight at 37 °C. After treatment, the cells were harvested and suspended in ice-cold ethanol (75%) and stored overnight at 4 °C. The fixed cells were then collected and washed with PBS. Cells were stained with 200 μL of PI (50 μg/mL) and 50 μL of RNAse A (100 μg/mL), and incubated at room temperature in the dark for 30 min. The cell cycle was analyzed using a FACScan flow cytometry system (Becton Dickinson, San Jose, CA, USA).

### 2.9. Statistical Analysis

Data are described as the mean ± standard deviation (SD). The statistical differences for target molecules such as IL-37, TNF-α, IL-6, collagenase-II, aggrecan, Ihh, MMP-13, caspase-3, caspase-9, Bax, poly(ADP-ribose) polymerase (PARP), or Bcl-2 mRNA expression between cells treated with IL-1β alone and IL-1β combined with IL-37 were measured using the nonparametric Mann–Whitney U-test. The differences in ROS production and apoptosis rate were also evaluated. A *p* value less than 0.05 was considered statistically significant. The statistical analyses and plots illustrated in figures were conducted using GraphPad Prism version 5.04 (San Diego, CA, USA).

## 3. Results

### 3.1. IL-37 Inhibits IL-1β-Induced Cytotoxicity in Rat Chondrocytes

**Figure 1:**
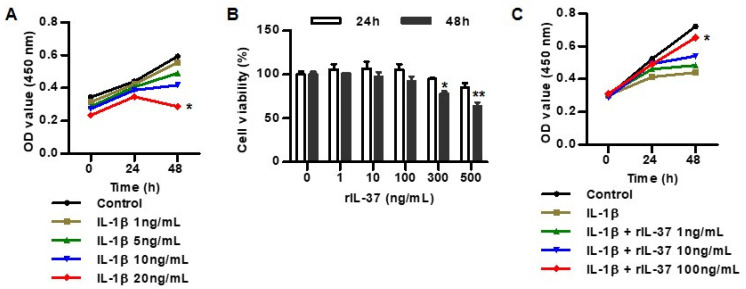
The effect of IL-37 on IL-1β-induced cytotoxicity in rat chondrocytes. (**A**) Cell viability in rat chondrocytes treated with variable concentrations of IL-1β (1, 5, 10, and 20 ng/mL) for 0 h, 24 h, and 48 h. CCK-8 assay was used for assessment of cytotoxicity in rat chondrocytes. * *p* < 0.05 compared to controls at 48 h. (**B**) Cell viability in rat chondrocytes treated with variable concentrations of rIL-37 (1, 10, 100, 300, and 500 ng/mL) for 24 h and 48 h. * *p* < 0.05 and ** *p* < 0.01 compared to 0 ng/mL of rIL-37 at 48 h. (**C**) Cell viability in 10 ng/mL of IL-1β-stimulated rat chondrocytes treated with rIL-37 for 0 h, 24 h, and 48 h. * *p* < 0.05 compared to IL-1β alone at 48 h. Data are presented as mean ± SD of three independent experiments.

We evaluated the cellular cytotoxicity of two cytokines, such as IL-1β and/or IL-37, on chondrocytes using a CCK-8 assay. Chondrocytes were treated with 1, 5, 10, and 20 ng/mL of IL-1β for 24 h and 48 h (Figure 1A). None of treatments with 1, 5, and 10 ng/mL of IL-1β for 24 h and 48 h significantly affected cellular proliferation, but treatment with 20 ng/mL of IL-1β for 48 h increased the cytotoxicity of the chondrocytes.

In the assessment of the cytotoxic effect of IL-37 on chondrocytes treated with different concentrations (1, 10, 100, 300, and 500 ng/mL) of rIL-37 for 24 h and 48 h, treatment with rIL-37 at 1, 10, and 100 ng/mL for 24 h and 48 h did not affect cellular toxicity in rat chondrocytes (Figure 1B). However, rIL-37 at 300 and 500 ng/mL for 48 h significantly increased chondrocyte cytotoxicity. In subsequent experiments, IL-1β was stimulated at 10 ng/mL for 48 h, and IL-37 was stimulated at 100 ng/mL for 24 h. In the measurement of the inhibitory effect of rIL-37 treatment on cytotoxicity using 10 ng/mL of IL-1β, we found that rIL-37 at 100 ng/mL attenuated the cytotoxicity of the chondrocytes (Figure 1C).

### 3.2. IL-37 Inhibits Inflammatory Cytokines and Extracellular Matrix Degradation in IL-1β-Mediated Rat Chondrocytes

**Figure 2:**
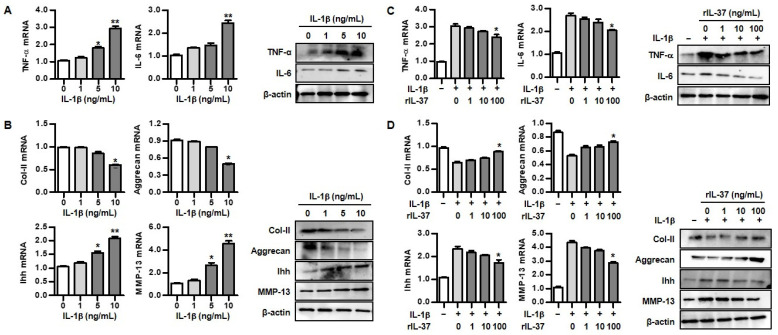
The effect of IL-37 on inflammatory cytokines and extracellular matrix degradation in IL-1β-mediated rat chondrocytes. (**A**) mRNA and protein expression of inflammatory cytokines by variable concentrations of IL-1β (1, 5, and 10 ng/mL) for 48 h in chondrocytes. * *p* < 0.05 and ** *p* < 0.01 compared to non-treated cells. (**B**) mRNA and protein expression of extracellular matrix molecules treated with variable concentrations of IL-1β (1, 5, and 10 ng/mL) for 48 h in chondrocytes. * *p* < 0.05 and ** *p* < 0.01 compared to non-treated cells. (**C**) mRNA and protein expression of inflammatory cytokines by different concentrations of rIL-37 (1, 10, and 100 ng/mL) in chondrocytes pretreated with IL-1β (10 ng/mL) for 48 h. * *p* < 0.05 compared to cells treated without rIL-37. (**D**) mRNA and protein expression of extracellular matrix molecules treated with different concentrations of rIL-37 (1, 10, and 100 ng/mL) in chondrocytes pretreated with IL-1β (10 ng/mL) for 48 h. * *p* < 0.05 compared to cells treated without rIL-37. Data are presented as mean ± SD of three independent experiments.

IL-1β is a crucial cytokine that is responsible for the regulation of inflammatory responses in OA. In the assessment of the expression of inflammatory cytokines, including TNF-α and IL-6, in IL-1β-induced rat chondrocytes, different concentrations of IL-1β (5 ng/mL and/or 10 ng/mL) resulted in significantly enhanced expression of TNF-α mRNA and IL-6 mRNA (Figure 2A). Similarly, IL-1β also increased production of TNF-α and IL-6 proteins. We assessed ECM degradation of rat chondrocytes stimulated by IL-1β. Those stimulated with IL-1β at 10 ng/mL suppressed the expression of collagen II and aggrecan mRNA. In contrast, IL-1β at 5 and 10 ng/mL significantly induced expression of Ihh and MMP-13 mRNA (Figure 2B). Production of collagen II and aggrecan by IL-1β was decreased in a dose-dependent manner, whereas IL-1β treatment increased Ihh and MMP-13 protein expression.

To confirm the role of the anti-inflammatory effects of IL-37, we evaluated whether IL-37 suppressed proinflammatory cytokines in IL-1β-stimulated chondrocytes. As shown in Figure 2C, chondrocytes stimulated with rIL-37 at 100 ng/mL attenuated the expression of TNF-α and IL-6 mRNA. In addition, TNF-α and IL-6 protein expression decreased in a dose-dependent manner when stimulated with rIL-37. Treatment with rIL-37 augmented collagen II, aggrecan mRNA, and protein expression in rat chondrocytes induced by IL-1β, but Ihh and MMP-13 mRNA, and protein expression, were markedly attenuated by the addition of rIL-37 (Figure 2D).

### 3.3. IL-37 Suppresses Mitochondrial ROS Generation in IL-1β-Stimulated Chondrocytes

**Figure 3:**
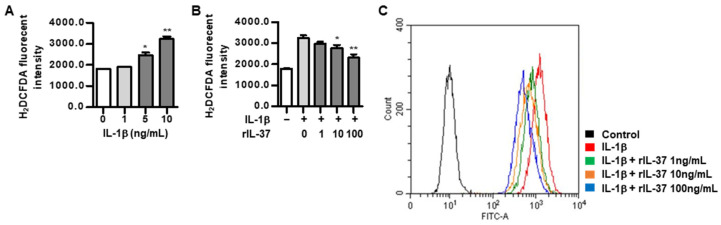
The effect of IL-37 on ROS generation in IL-1β-stimulated chondrocytes. (**A**) ROS generation by variable concentrations of IL-1β (1, 5, and 10 ng/mL) for 48 h in chondrocytes. * *p* < 0.05 and ** *p* < 0.01 compared to non-treated cells. DCFDA cellular ROS assay was used for measurement of ROS generation in rat chondrocytes. (**B**) ROS generation by different concentrations of rIL-37 (1, 10, and 100 ng/mL) in chondrocytes pretreated with IL-1β (10 ng/mL) for 48 h. * *p* < 0.05 and ** *p* < 0.01 compared to cells treated without rIL-37. (**C**) Detection of ROS using flow cytometry in the stimulation with different concentrations of rIL-37 (1, 10, and 100 ng/mL) in chondrocytes pretreated with IL-1β (10 ng/mL) for 48 h. Data are presented as mean ± SD of three independent experiments.

We assessed the role of IL-37 in mitochondrial oxidative stress in IL-1β-stimulated chondrocytes. As shown in Figure 3A, IL-1β at concentrations of 5 ng/mL and 10 ng/mL increased ROS generation, compared to non-stimulated chondrocytes. In contrast, increased intracellular ROS generation stimulated by IL-1β was markedly reduced in chondrocytes in response to 10 ng/mL and 100 ng/mL of rIL-37 (Figure 3B). ROS, as measured by a fluorescent probe incubated with IL-1β, were significantly attenuated by rIL-37 stimulation in a dose-dependent manner (Figure 3C).

### 3.4. IL-1β Induced Apoptosis in Rat Chondrocytes

**Figure 4:**
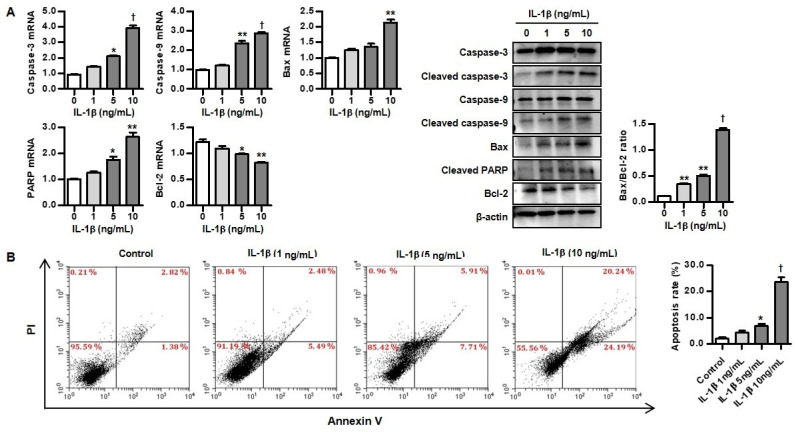
The stimulatory effect of IL-1β on chondrocytes apoptosis. (**A**) mRNA and protein expression of apoptosis-related molecules by variable concentrations of IL-1β (1, 5, and 10 ng/mL) for 48 h in chondrocytes. * *p* < 0.05, ** *p* < 0.01, and ^†^ *p* < 0.001 compared to non-treated cells. (**B**) Representative FACS scatter plots of annexin V/propidium iodide staining for treatment with variable concentrations of IL-1β (1, 5, and 10 ng/mL) for 48 h in chondrocytes. Bar plot for apoptosis rates (%) was presented. * *p* < 0.05 and ^†^ *p* < 0.001 compared to controls. Data are presented as mean ± SD of three independent experiments.

In the assessment of cellular apoptosis in IL-1β-induced chondrocytes, IL-1β stimulation significantly induced pro-apoptotic markers such as caspase-3, caspase-9, Bax, and PARP mRNA and protein expression, but suppressed the anti-apoptotic marker Bcl-2 (Figure 4A). The Bax/Bcl-2 ratio was dose-dependently increased in rat chondrocytes stimulated with IL-1β. As shown in Figure 4B, annexin V-FITC/PI double-staining and flow cytometry indicated that the apoptosis rate of chondrocytes (6.9 ± 0.6% and 23.6 ± 1.6%) treated with 5 ng/mL and 10 ng/mL of IL-1β was significantly higher, compared to that of control chondrocytes (2.0 ± 0.4%), respectively. IL-1β is therefore responsible for inhibiting chondrocyte differentiation, and for promotion of chondrocyte apoptosis.

### 3.5. IL-37 Inhibits IL-1β-Induced Chondrocyte Apoptosis

**Figure 5:**
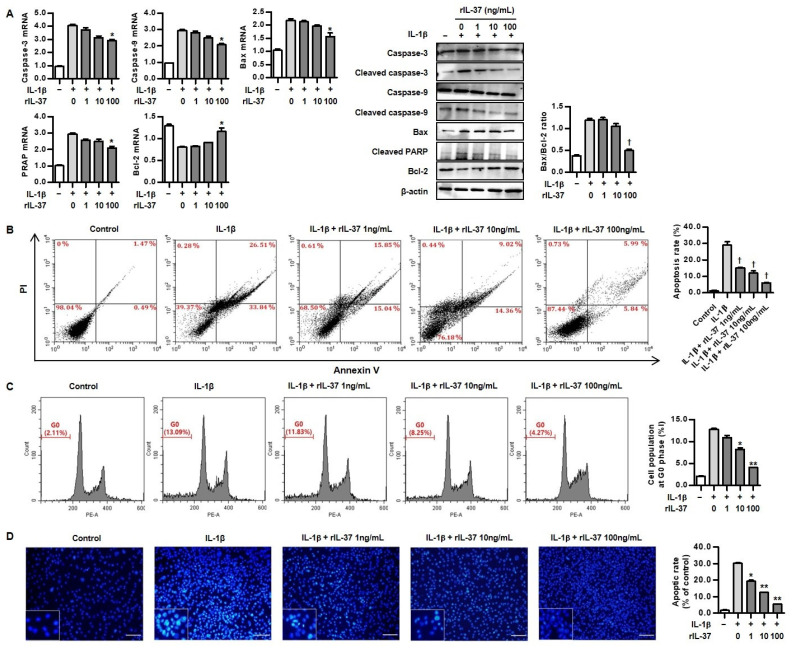
The effect of IL-37 on IL-1β-mediated chondrocyte apoptosis. (**A**) mRNA and protein expression of apoptosis-related molecules by different concentrations of rIL-37 (1, 10, and 100 ng/mL) in chondrocytes pretreated with IL-1β (10 ng/mL) for 48 h. * *p* < 0.05 and ^†^ *p* < 0.001 compared to cells treated without rIL-37. (**B**) Representative FACS scatter plots of annexin V/propidium iodide staining for treatment with different concentrations of rIL-37 (1, 10, and 100 ng/mL) in chondrocytes pretreated with IL-1β (10 ng/mL) for 48 h. Bar plot for apoptosis rates (%) was presented. ^†^ *p* < 0.001 compared to IL-1β alone. (**C**) The proportion of chondrocytes at G0 phase in the treatment with 1, 10, and 100 ng/mL of rIL-37 in chondrocytes pretreated with IL-1β (10 ng/mL) for 48 h using cell cycle analysis. * *p* < 0.05 and ** *p* < 0.01 compared to IL-1β alone. (**D**) Representative Hoechst 33342 staining for apoptosis in chondrocytes by different concentrations of rIL-37 (1, 10, and 100 ng/mL) in chondrocytes pretreated with IL-1β (10 ng/mL) for 48 h. * *p* < 0.05 and ** *p* < 0.01 compared to IL-1β alone. Scale bar = 100 μm. Data are presented as mean ± SD of three independent experiments.

With respect to the effect of IL-37 on chondrocyte apoptosis, rIL-37 treatment inhibited expression of cellular pro-apoptotic markers such as caspase-3, caspase-9, Bax, and PARP, but it increased anti-apoptotic markers (Figure 5A). Furthermore, 100 ng/mL of rIL-37 treatment significantly decreased the Bax/Bcl-2 ratio, suggesting an anti-apoptotic effect of IL-37. A flow cytometry assay indicated that apoptosis of rat chondrocytes treated with 1, 10, and 100 ng/mL of rIL-37 was decreased by 15.2 ± 0.2%, 11.9 ± 1.4%, and 6.1 ± 0.1%, respectively, compared to chondrocytes stimulated with 10 ng/mL of IL-1β alone (29.0 ± 2.0%) (Figure 5B). Cell cycle analysis was performed to measure early apoptosis of cells treated with 10 ng/mL of IL-1β with or without the addition of 1, 10, and 100 ng/mL of rIL-37 (Figure 5C). The result showed that the population of apoptotic cells at the G0 phase was markedly increased in rat chondrocytes stimulated with 10 ng/mL of IL-1β alone, compared to that of the control (12.9 ± 0.3% vs. 2.1 ± 0.1%). However, the addition of 10 and 100 ng/mL of rIL-37 in IL-1β-induced chondrocytes significantly reduced the population of apoptotic cells (8.3 ± 0.6% and 4.1 ± 0.2%, respectively), but 1 ng/mL of rIL-37 (10.9 ± 0.9%) did not. The morphological changes of chondrocytes stained with Hoechst 33342 are shown in Figure 5D. The number of condensed nuclei stained with Hoechst 33342 and induced with IL-1β gradually decreased after stimulation with rIL-37 at concentrations of 1, 10, and 100 ng/mL, suggesting that stimulation with rIL-37 markedly reduces the number of apoptotic chondrocytes.

## 4. Discussion

The pathogenesis of OA is complex, and is now understood to involve several components, including mechanical, metabolic, genetic, and inflammatory factors [1,2,3]. Inflammation of affected joints is believed to take place even in the early phases of the development and progression of OA. Given the role of inflammatory cytokines in OA, IL-1β, which is highly expressed in synovial tissue, cartilage, and subchondral bone, in addition to TNF-α and IL-6, is a crucial mediator responsible for ECM degradation, and recruitment of other inflammatory molecules involved in the progressive destruction and inflammatory response of articular cartilage in OA [10,11,12,13]. In this study, we found that IL-1β markedly induced other proinflammatory cytokines, and promoted the degradation of the ECM of chondrocytes by regulating ROS-mediated chondrocyte apoptosis. Based on the crucial role of inflammatory cytokines in the development of OA, some human and animal studies provided promising evidence that IL-1 blocking strategies, such as anakinra (a recombinant human IL-1 receptor antagonist, IL-1Ra) or gene therapy using IL-1Ra, were well-tolerated, improved OA-related symptoms, such as pain, stiffness, and limitation of motion, and reduced the histologic severity of cartilage breakdown [23,24,25,26]. We found that IL-37, an anti-inflammatory cytokine, had an inhibitory effect on IL-1β-mediated inflammatory and pro-apoptotic actions in the pathogenesis of OA. This suggests that IL-37 can be an effective anti-cytokine therapeutic strategy for OA.

It is well-established that mitochondria are crucial in regulating cell life and death [27]. Especially, the diverse action mechanisms of mitochondria as the orchestrators of apoptosis have come to be understood, including the change in cellular redox potential, alternation in electron transport, and the release of caspase protease-activating proteins. Mitochondria play a critical role in diverse cellular function and survival in aging-related diseases, such as OA. Mitochondrial dysfunctions can contribute to the pathogenic pathways of development and progression in OA through increased intracellular ROS levels, because mitochondria are a main source of ROS generation [28,29]. Increased ROS production sequentially induces oxidative stress, which can disrupt cartilage homeostasis through diverse pathogenic mechanisms, such as chondrocyte apoptosis, degradation of ECM, and cytokine-mediated inflammation [30,31]. Well-characterized and potent ROS in chondrocytes are superoxide and hydrogen peroxide, which are catalyzed from the conversion of superoxide and hydrogen peroxide by superoxide dismutase (SOD). A proteomic study demonstrated lower SOD concentrations with mitochondrial antioxidant properties and increased intracellular ROS levels, compared with those of normal chondrocytes in human OA chondrocytes [32]. SOD2 depletion in chondrocytes induced oxidative stress and damage, indicating a pathogenic role for ROS in the disturbance of cartilage homeostasis in OA [33]. In addition, Zhou et al. reported that treatment with isorhamnetin could inhibit chondrocyte apoptosis by suppressing ROS generation in OA [34]. We also found that chondrocytes stimulated with IL-1β increased the release of intracellular ROS, which were significantly suppressed by rIL-37 treatment of chondrocytes. ROS is therefore a powerful mediator of cellular signaling pathways involved in the pathogenesis of OA, including chondrocyte apoptosis and ECM degradation.

Chondrocytes are only resident cells in articular cartilage, playing a crucial role in the maintenance of ECM. Chondrocyte survival is responsible for the repair and maintenance of proper articular cartilage through assembling the ECM in response to traumatic injury or possibly inflammatory conditions [7]. Recently reported evidence has revealed a close association between cartilage degradation and chondrocyte apoptosis in the pathogenesis of OA [6,7]. At the advanced late stage, OA is characterized by having a reduced number of chondrocytes, often combined with empty lacuna, which lead to the failure of regeneration of articular cartilage structures. This implies that chondrocyte apoptosis is responsible for the alterations of cartilage architecture in OA. Diverse stimuli could induce chondrocyte apoptosis with DNA damage, cytokines, toxins, irradiation, or hypoxia as internal inducers, and Fas ligand, TNF-related apoptosis-inducing ligand (TRAIL), or TNF as death ligands [5,6,7].

Human articular chondrocytes stimulated with IL-1 (0.1 to 10 ng/mL) as an inducer of endogenous nitric oxide (NO) synthesis did not trigger cellular apoptosis, in addition to TNF, INF-γ, lipopolysaccharide (LPS), or their combinations as different NO inducers, whereas exogenous NO derived from sodium nitroprusside and s-nitro-N-acetyl-D-L-penicillamine as NO donors significantly induced morphological apoptotic changes in chondrocytes [35]. However, oxygen radicals generated from chondrocytes stimulated with IL-1 do not appear to trigger apoptotic changes. Similarly, Clancy et al. showed that an increased NO release in bovine chondrocytes treated with TNF and IL-1 did not directly influence cell viability [36]. In contrast, IL-1β was shown to be anti-apoptotic through an NF-κB-mediated protective mechanism in human articular chondrocytes [37]. IL-1-mediated chondrocyte apoptosis is currently subject to debate. Contrary to previous research results, we found that IL-1β-mediated ROS production could induce the apoptosis of rat articular chondrocytes through the mitochondrial pathway. In addition, an increase in ROS levels was found to be related to enhanced ECM degradation. Similarly, Zhuang et al. demonstrated that an experimental stress model incubated in H_2_O_2_ led to caspase-dependent and caspase-independent apoptosis of rat chondrocytes [38]. Another study using an OA mouse model showed that aucubin, a natural compound isolated from *Aucuba japonica*, protected articular cartilage from IL-1β-mediated chondrocyte apoptosis by blocking intracellular ROS production [39]. It implicated that IL-1β may play a role as a proinflammatory cytokine with pro-apoptotic properties in the pathogenesis of OA.

Aberrant proinflammatory cytokines originating from resident cells of affected joints have crucial roles as signaling molecules in the immune response to cartilage damage, bone remodeling, and synovial inflammation in OA [10,11,12,13]. Data from recent experimental studies provided accumulating evidence that IL-37 might be helpful in controlling OA-related symptoms and signs [18,19,20]. Stimulation with IL-1β or OA synovium-conditioned medium in human mesenchymal stem cells markedly suppressed sulfated glycosaminoglycan (sGAGs) production, which was significantly reversed by IL-37 [20]. IL-37 also enhanced the collagen II/I ratio and inhibited the gene expression of other proinflammatory cytokines such as IL-1β, IL-6, and IL-8 [18,20]. It implicated that IL-37 appears to play a role in promoting the recovery of impaired cartilage and the microinflammatory environment in a damaged OA joint. We consistently found that IL-37 inhibited the proinflammatory cytokines IL-6 and TNF-α in IL-1β-stimulated chondrocytes. In the assessment of the protective effect of IL-37 on proteoglycan loss, van Geffen et al. observed a stimulatory effect of IL-37 on the release of sGAGs, and an inhibitory effect on MMP-3 and MMP-13 expression involved in cartilage degradation associated with human OA chondrocytes [18,19]. We also found that IL-1β increased gene and protein expression of proteolytic enzyme MMP-13, but both were suppressed by IL-37. Based on these observations, IL-37 induced by IL-1β plays a crucial role in protecting cartilage degradation by blocking the release of proinflammatory cytokines and proteolytic catabolic enzymes.

Evidence for the protective effect of IL-37 on diverse inflammatory conditions through attenuating cellular apoptosis of disease-related target cells or tissues has been accumulating in inflammatory conditions such as acute respiratory distress syndrome (ARDS), diabetic nephropathy, and atherosclerosis [40,41,42]. In the LPS-induced ARDS animal model, a TUNEL staining assay showed that the number of apoptotic cells after LPS injection was markedly reduced following IL-37 treatment [40]. In addition, increased levels of the pro-apoptotic biomarkers, Bax and cleaved caspase3, by LPS injection were reversed by IL-37. Endothelial dysfunction of cardiovascular diseases is caused by initiation of atherosclerosis in response to vascular inflammation. Considering the protective role of IL-37 in endothelial cell apoptosis in an endothelial dysfunction model, IL-37 treatment significantly decreased the number of apoptotic cells in an in vitro model using human umbilical vein endothelial cells treated with oxidized low-density lipoprotein [41]. The beneficial mechanism of anti-apoptotic IL-37 was found to be promoted by enhanced autophagy. Zhang et al. demonstrated a significantly decreased IL-37 level and increased apoptosis in podocytes treated with high concentrations of glucose [42]. In addition, IL-37 inhibited apoptosis of podocytes with high levels of glucose by blocking the STAT3-cyclophilin A signaling pathway. However, few data about the role of IL-37 in chondrocyte apoptosis in the impairment of cartilage formation in OA are available. We found that IL-1β induced apoptosis by activating the mitochondrial apoptotic pathway and generating ROS in chondrocytes, effects that were significantly alleviated by IL-37. These findings indicate that IL-37 can improve impaired cartilage formation depending on the inhibition of chondrocyte apoptosis.

## 5. Conclusions

**Figure 6:**
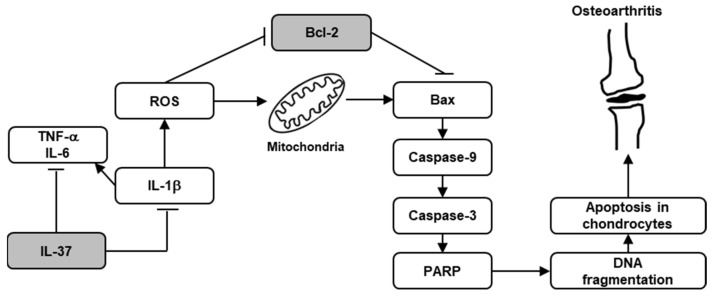
Overview of protective effect of IL-37 on IL-1β-induced chondrocyte apoptosis. IL-1β stimulated mitochondrial ROS generation in chondrocytes, which triggered chondrocyte apoptosis through the mitochondrial apoptosis signal pathway. However, IL-37 treatment inhibited chondrocyte apoptosis induced by IL-1β via inhibition of ROS generation and blockage of the mitochondrial apoptosis pathway.

This study revealed that IL-1β markedly increased the apoptosis rate of rat chondrocytes by ROS generation via the mitochondrial apoptosis pathway (Figure 6). It suggests that apoptotic chondrocytes, through the production of ROS mediated by inflammatory cytokines, and IL-1β in particular, might be an important pathological mechanism in the development of OA. We found beneficial effects of IL-37 on the degradation of ECM and cellular apoptosis of IL-1β-induced rat chondrocytes in OA. Based on these findings, IL-37 may prove to be a novel therapeutic option against apoptosis of chondrocytes in OA.

## Data Availability

The data underlying this article will be shared on reasonable request to the corresponding author.
